# Communication methods and production techniques in fixed prosthesis fabrication: a UK based survey. Part 2: Production techniques

**DOI:** 10.1038/sj.bdj.2014.644

**Published:** 2014-09-26

**Authors:** J. Berry, M. Nesbit, S. Saberi, H. Petridis

**Affiliations:** 1 Clinical Lecturer, Department of Adult Oral Health, Institute of Dentistry, Barts and The London School of Medicine and Dentistry, Queen Mary University of London, London; 2 Senior Technical Instructor, Prosthodontic Unit, UCL Eastman Dental Institute, London; 3 Senior Lecturer, Department of Restorative Dentistry, Prosthodontics Unit, UCL Eastman Dental Institute, London.

## Abstract

**Aim** The aim of this study was to identify the communication methods and production techniques used by dentists and dental technicians for the fabrication of fixed prostheses within the UK from the dental technicians' perspective. This second paper reports on the production techniques utilised.

**Materials and methods** Seven hundred and eighty-two online questionnaires were distributed to the Dental Laboratories Association membership and included a broad range of topics, such as demographics, impression disinfection and suitability, and various production techniques. Settings were managed in order to ensure anonymity of respondents. Statistical analysis was undertaken to test the influence of various demographic variables such as the source of information, the location, and the size of the dental laboratory.

**Results** The number of completed responses totalled 248 (32% response rate). Ninety percent of the respondents were based in England and the majority of dental laboratories were categorised as small sized (working with up to 25 dentists). Concerns were raised regarding inadequate disinfection protocols between dentists and dental laboratories and the poor quality of master impressions. Full arch plastic trays were the most popular impression tray used by dentists in the fabrication of crowns (61%) and bridgework (68%). The majority (89%) of jaw registration records were considered inaccurate. Forty-four percent of dental laboratories preferred using semi-adjustable articulators. Axial and occlusal under-preparation of abutment teeth was reported as an issue in about 25% of cases. Base metal alloy was the most (52%) commonly used alloy material. Metal-ceramic crowns were the most popular choice for anterior (69%) and posterior (70%) cases. The various factors considered did not have any statistically significant effect on the answers provided. The only notable exception was the fact that more methods of communicating the size and shape of crowns were utilised for large laboratories.

**Conclusion** This study suggests that there are continuing issues in the production techniques utilised between dentists and dental laboratories.

## Introduction

Prosthodontics is a discipline that requires a synergy between the dentist and dental technician, in order to fabricate intraoral prostheses with acceptable fit, function and aesthetics.^1^,^2^,^3^ The General Dental Council's (GDC) policy document *Principles of dental team working*^4^ states that 'members of the dental team should work effectively together in patients' best interest'. In addition, legislation such as the updated European Medical Devices Directive^5^ must be complied with so that all laboratory made products are constructed of materials considered to be safe and to a standard that will not harm the patient in any way. In order for these recommendations to be implemented, both dentists and dental technicians need to be aware of the processes and protocols used in the fabrication of fixed prostheses.

However, a number of studies^6^,^7^,^8^,^9^,^10^,^11^,^12^,^13^ from around the world have highlighted the need for improved communication methods and production techniques between dentists and dental technicians during fabrication of fixed restorations. Problems have been identified in various parts of production processes and communication ranging from quality of impressions, to adequate tooth preparation, articulation, and adequate instructions regarding the use of materials.^6^,^7^,^8^,^9^,^10^,^11^,^12^,^13^

Undergraduate training should theoretically prepare dentists with the required knowledge to provide fixed prostheses in a safe and predictable manner. However, a number of studies^12^,^14^ have raised concerns regarding the competency of newly qualified dentists on their understanding of production techniques, possibly due to the reduction in dental technology teaching within the undergraduate curriculum,^1^,^15^ as well as the lack of interaction between dental technicians and students during these important formative years. This apparent disparity has led to the conclusion^12^ that the General Dental Council has failed in its aims published in *The first five years: a framework for undergraduate dental education*.^16^ Indeed in Australia this is now being addressed with the introduction of inter-professional teaching schemes.^17^

The purpose of this cross-sectional study was to identify the communication methods and production techniques used by dentists and dental technicians for the fabrication of fixed prostheses within the UK from the dental technicians' perspective. Part one of this cross-sectional survey reported on the communication issues between dentists and dental laboratories.^13^ The current publication concentrates on the production techniques used for fixed prosthesis fabrication.

## Materials and methods

The details regarding materials and methods have been published in the first paper.^13^ A questionnaire was constructed to investigate communication methods and production techniques used between dentists and dental laboratories from the laboratories perspective. The final questionnaire consisted of 30 questions within the following subcategories: general information, communication methods, impression disinfection and suitability, production techniques, shade matching, and time and team management issues.

The Dental Laboratories Association (DLA, Nottingham, UK) was approached and approved the use of their database of e-mail contacts (782 addresses). A web-based survey tool, Opinio (ObjectPlanet Inc. Oslo, Norway), was utilised for the administration of the survey and assimilation of data. Settings were managed in order to ensure anonymity of respondents.

The data collected was presented as descriptive statistics and analysed using Fisher's exact test, the Mann-Whitney test or the Spearman's rank correlation (SPSS 12.0; SPSS Inc, Chicago). P-values of less than 0.025 were regarded as statistically significant. A significance level of 2.5% was chosen rather than the conventional 5% to avoid spuriously significant results arising from multiple testing.

The null hypothesis was that factors such as the source of information used to answer the questionnaire, the location, and size of the dental laboratory, did not influence the communication methods and production techniques.

## Results

The number of responses totalled 248, which yielded a 32% response rate. Sixty-eight respondents answered only some of the questions. The results presented in this paper pertain to the subchapters of general information, disinfection and suitability of impressions and production techniques. The subchapters and questions along with the results in parentheses are depicted in Table 1.

The results of the general information subchapter have been published in part one,^13^ but the main points are presented here as they were factors for the statistical analysis that followed. The majority of the information (81%) used to answer the survey questions were sourced from memory. Ninety percent of the respondents were based in England. This unequal distribution among England, Scotland, Wales and Northern Ireland did not permit any further analysis of this particular factor. The majority of dental laboratories were categorised as small sized (43% working with up to 25 dentists), 38% as medium (working with 26-75 dentists) and 19% as large (working with 76+ dentists).

The results of this study showed that a significant number of respondents (52%) considered that less than half of the impressions received from the dentist were clearly labelled as having been disinfected. Sixty-five percent of dental laboratories indicated that they routinely disinfected the impressions received from the dentist before pouring them up.

The most popular impression tray used in the fabrication of crowns and bridgework was the full arch plastic tray, which was used in 61% and 69% of cases respectively. Custom made trays were only used in 10% of cases and quadrant plastic trays were the least popular. A significant number of respondents (17%) considered that the majority of final master impressions received were of poor quality and inadequate to use for a varied number of reasons including air voids, defects at the preparation margin and deformation of the impression material.

The aforementioned results, pertaining to the disinfection and suitability of impressions, were not influenced by the size of the laboratory or the source of information with the exception of the responses about the inadequacy of the master impressions (p = 0.03), which suggested that the proportion of inadequate impressions was greater in the records group than the memory group.

Regarding the adequacy of tooth preparations, the results of this study showed that, on average, 18% of respondents considered that they routinely received tooth preparations where there had been inadequate bucco-lingual tooth reduction. The analysis showed that the percentage was statistically (p = 0.01) higher (28%) in the records group compared to the memory group. With respect to occlusal tooth reduction, 26% indicated that they frequently encountered tooth preparations with insufficient reduction.

The semi-adjustable articulator was the most frequently used (44%) in the fabrication of fixed prostheses followed by the simple hinge type (28%). This survey indicated that only 11% of the dental laboratories perceived that the majority of inter-occlusal records were accurate.

**Figure 1:**
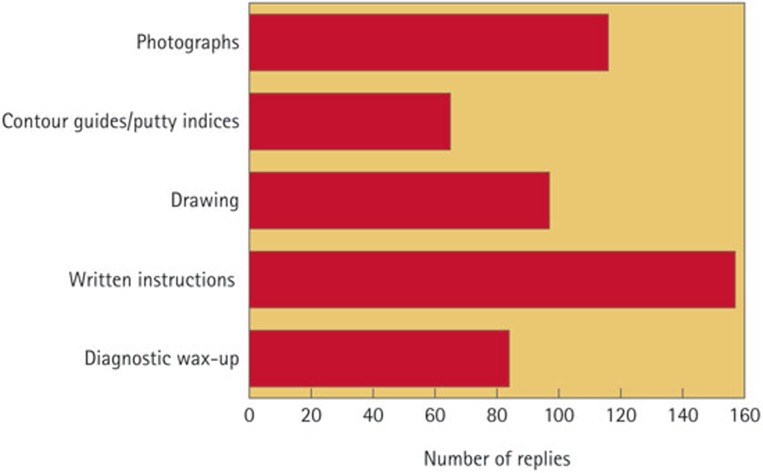
Communication of the size and shape of the crown

The majority of respondents (68%) reported that they rarely received any particular guide, such as a diagnostic wax-up, or impressions from provisional restorations, in order to communicate the shape and size of the definitive restoration. Written instructions were the most widely used means of communicating the size and shape of crowns, and were often supplemented with photographs, drawings, or the use of diagnostic wax-ups (Fig. 1). The statistical analysis revealed that the size of the laboratory affected these communication methods as they all varied significantly between groups; the diagnostic wax-up (p = 0.002), contour guides (p = 0.02) and photographs (p = 0.01). These three methods of communication were least common within the small-sized laboratory group. The use of written instructions and drawings was not found to be significantly associated with the size of the laboratory.

The study also showed that dental technicians often had to decide on the type of material and the surface on which to use the material, as it had not been accurately prescribed by the dentist. Almost a quarter (24%) of dental technicians had to routinely choose both for the materials to be used for the fixed prostheses as well as the particular surfaces that needed to be covered with an aesthetic veneering material.

**Figure 2:**
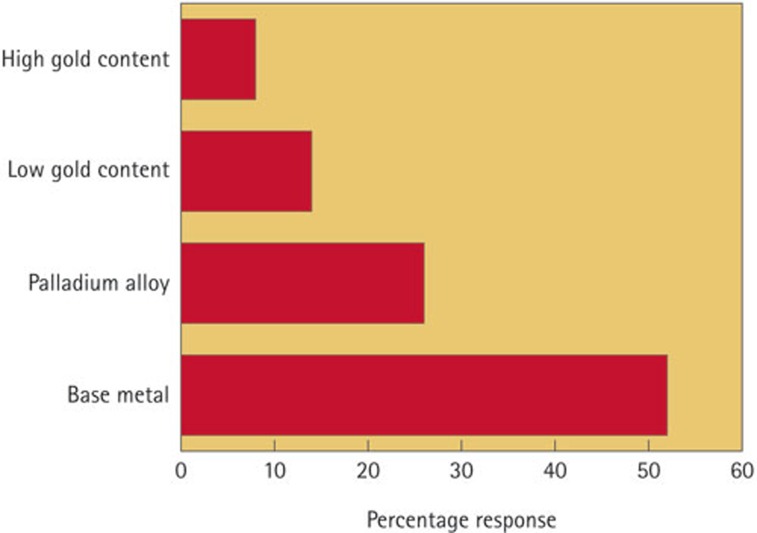
Most commonly used metal alloys

For the fabrication of fixed prostheses, base metal alloys (52%) were the most commonly used, with high gold content alloys only used in 8% of cases (Fig. 2).

The most commonly requested combination of materials for the construction of both anterior and posterior crowns was metal-ceramic (69% and 71% respectively). All-ceramic crowns accounted for 29% of anterior cases and only 8% of posterior crowns. Metal-only posterior crowns were only used in 19% of situations. No significant statistical observations were noticed for the aforementioned results.

## Discussion

This cross-sectional survey was undertaken to identify the communication and production techniques used by dentists and dental technicians for the fabrication of fixed prostheses within the UK from the dental technicians' perspective. The current publication reports on the production techniques used. The response rate of 32% was similar to previously published surveys^10^,^11^,^12^,^18^ of dental laboratories. This current survey was unique in that it was administered online in the anticipation of making it more appealing and easier to participate.^13^ However, the response rate was similar to previous postal ones. This survey was limited by the fact that no distinction was made between possible differences in production techniques in laboratories, which provided a fully private service, a fully NHS service or a mixed arrangement.^6^,^13^

Personal bias may have affected the accuracy of the results as the majority of the information used to answer the survey questions was sourced from memory. Dental technicians could have exaggerated the extent of poor impression disinfection and suitability of the impressions, as well as potential issues in production techniques. Nevertheless, the statistical analysis showed that the source of the information did not play a significant role.

The results of this study showed that a significant number of dental laboratories were receiving impressions from dentists that were not clearly labelled as having been disinfected. It was also shown that 65% of laboratories would routinely disinfect the impressions on their arrival. It seems that there is a lack of agreement between dentists and laboratories regarding decontamination and disinfection of dental impressions, even though clear guidelines have been made available via the British Dental Association.^19^ These results are in agreement with previous studies in the UK,^20^ Greece,^11^ and the USA.^21^ Robust disinfection protocols are essential to prevent the risk of cross infection between team members.^22^,^23^,^24^

Full-arch plastic trays were the most frequently used impression trays for the fabrication of crowns and bridgework, and this confirms previous findings.^6^,^11^,^25^ Dual arch impression trays were the second most popular (14%) for the fabrication of single crowns, and were also used in 7% of bridgework cases. This technique has become more popular with dentists as a time and material efficient way of recording an impression.^26^,^27^,^28^ However, the available literature^29^,^30^ shows that dual-arch impressions only compare favourably with full-arch impressions with respect to the fabrication of single units. Dental laboratories considered this technique as being difficult to work with, leading to the possibility of further inaccuracies. Custom made trays were only prescribed in 9-11% of cases, which is low considering that the master impression technique recommended by the British Society for Restorative Dentistry^31^ is the utilisation of a custom tray with a medium body silicone within the tray, and a low viscosity silicone syringed around the tooth preparation.

A concerning finding of this survey was that a high proportion of final master impressions were considered as inadequate for use by the dental technician. Most troublesome was the fact that the majority of the inadequate impressions presented with a combination of problems. Similar results regarding the frequency and aetiology of inadequate definitive impressions have been reported in previous studies^7^,^11^,^21^,^32^,^33^,^34^ and the lack of improvement is a troublesome issue. It is essential that the dentist carefully scrutinises the impression, preferably under good lighting and magnification, to ensure its suitability before sending it to the laboratory.

The lack of sufficient tooth preparation presents the dental technician with the difficult task of fabricating a crown or bridge with adequate form and aesthetics.^35^ This study confirmed the results of previous ones^11^,^36^,^37^,^38^ in that under preparation of teeth frequently happens, and this is the first time that it has been reported in the UK. The routine clinical use of preparation guides, such as putty or plastic indices derived from diagnostic wax-ups would help ensure the correct occlusal and bucco-lingual tooth reduction.

To date there has been no research data on the use of articulators and occlusal records within commercial dental laboratories the UK. This particular survey showed that the semi-adjustable articulator was favoured in the fabrication of fixed crown and bridgework, being used 44% of the time. This type of articulator is also preferred in dental schools in the UK^39^ and is the preferred choice of the British Society of Restorative Dentistry.^31^ A troublesome finding was the use of static articulators in 9% of cases. These types of articulators are not indicated for any quality restorative work. Interestingly, the results of this study showed that only 11% of occlusal records received were routinely considered adequate. The majority of records sent to the technicians were probably not used and discarded. An accurate and usable occlusal record is very important and any inaccuracies may lead to the need for extensive intraoral adjustments, which may compromise aesthetics or mechanical strength of restorations.^40^,^41^ A previous study in Greece^11^ reported that dental laboratories had confidence in the jaw registration records provided and may be a reflection on possible different jaw registration techniques taught and used by dentists in the UK and Greece.

The results of this study also showed that, in the majority of cases (68%), no guides were provided by the dentists for the fabrication of definitive prostheses. In cases that it happened, it was usually in the form of written instructions or photographs. Guides, such as the diagnostic wax-up, a copy of the provisional crowns, and occlusal aids, such as a custom incisal guide table should be provided by dentists.^41^ The statistical analysis showed that diagnostic wax-ups, contour guides, and photographs were used more often with large labs and this may reflect the need for improved communication in such settings.

This survey concurs with previous ones^6^,^25^,^42^ that dentists commonly do not prescribe the materials to be used or the surfaces to be covered by the veneering material in the construction of crowns or bridgework, leaving the decision to the technician. The dentist is now obliged by law to prescribe the materials to be used,^4^,^5^ and it is the responsibility of the dentist to assess each patient individually to decide on the surfaces to be covered with a particular material.

The increasing cost of gold was reflected in the popular use (52%) of base metal alloys for crown and bridgework. This has also been a trend in other countries,^7^,^11^ but based on the previous answer, it would be interesting to explore whether the dentists are aware, as they should be, of the types of metal alloys that are used for the fabrication of the prescribed fixed prostheses. The increased use of base metal alloys may have implications regarding the corrosion resistance of crown and bridgework.^43^ Finally the survey investigated the combination of materials used in certain situations. Metal-ceramic crowns were still the most popular choice for anterior and posterior crowns. Many previous studies^44^,^45^ have reported their good survival rates. All-ceramic crowns, despite having potentially high survival rates,^46^,^47^ were still not frequently prescribed by dentists, even for anterior cases. Surprisingly, full metal coverage posterior crowns were not a popular choice nowadays, probably a reflection of the increased cosmetic awareness of the population in general.

## Conclusions

Within the limitations of this UK based study, the following conclusions could be drawn:

1. There is still an apparent lack of protocol in the disinfection of impressions between the dentists and laboratories, thus creating a potential health risk
2. Plastic full arch trays were the dentists' preferred choice of impression tray for recording master impressions
3. Dentists frequently sent master impressions to the laboratory, which are not appropriate for the fabrication of fixed prostheses
4. More use of diagnostic wax-ups and reduction guides to ensure adequate tooth removal should be used by dentists
5. The dental technicians in the main did not trust the authenticity of the occlusal relationship records provided
6. Dentists frequently failed to prescribe the material to be used or the design of the prosthesis, incorrectly leaving the decision to the dental technician
7. Metal-ceramic crowns were still the most popular choice for both anterior and posterior units.

## Figures and Tables

**Table 1:** Relevant subchapters of the questionnaire with answers in parentheses

**GENERAL INFORMATION**1. Please indicate the source of the information that you will be giving:From memory (81%) From records (19%)2. This survey is anonymous so please indicate the country that you are based in:England (90%) Scotland (4%) Northern Ireland (1%) Wales (5%)3. Approximately, what number of dentists do you currently work with?1 – 25 (43%) 26 – 50 (30%) 51 – 75 (8%) 76 – 100 (6%) 100+ (13%)**IMPRESSION DISINFECTION AND SUITABILITY**4. When the impressions arrive at the dental laboratory, what percentages are clearly labelled indicating that they have been disinfected?0-25% (30%) 26-50% (22%) 51-75% (22%) 76-100% (26%)5. Do you always disinfect the impressions from the dentists before pouring them up?Yes (65%) No (35%)**PRODUCTION TECHNIQUES**6. What is the most popular impression tray used for the fabrication of crowns?Dual arch impression -triple tray technique (14%) Quadrant plastic trays (9%) Quadrant metal trays (1%) Full arch plastic trays (61%) Full arch metal trays (6%) Custom tray (9%)7. What is the most popular impression tray used for the fabrication of bridgework that you see in your laboratory?Dual arch impression - triple tray technique (7%) Quadrant plastic trays (4%) Quadrant metal trays (0%) Full arch plastic trays (69%) Full arch metal trays (9%) Custom tray (11%)8. What is the approximate percentage of final master impressions that you consider to be inadequate?0-25% (57%) 26-50% (26%) 51-75% (12%) 76-100% (5%)9. What is the main reason for the poor quality of the final master impression?Presence of bubbles/voids (8%) Deformation of impression material (11%) Defects at the preparation margins (7%) Combination of above (72%) Other reasons (2%)10. How often do you feel that there has been insufficient bucco-lingual tooth removal to achieve satisfactory crown fabrication?0-25% (48%) 26-50% (34%) 51-75% (16%) 76-100% (2%)11. How often do you feel that there has been insufficient occlusal tooth removal to achieve satisfactory crown fabrication?0-25% (43%) 26-50% (31%) 51-75% (20%) 76-100% (6%)12. What type of articulator do you usually use for fabrication of fixed crown and bridgework?Static - only up & down motion (9%) Simple hinge (28%) Mean value (19%) Semi adjustable (44%)13. What percentage of dentists send you the appropriate occlusal records?0-25% (33%) 26-50% (29%) 51-75% (27%) 76-100% (11%)14. What percentage of dentists provide you with a guide to aid you in the fabrication of the definitive prosthesis (diagnostic wax up, tooth preparation guides, impression of provisional restorations)?0-25% (68%) 26-50% (23%) 51-75% (4%) 76-100% (5%)15. How do dentists communicate with you the shape and size of the crown? (Tick all that apply)Diagnostic wax-up (16%) Written instructions (30%) Drawing (19%) Contour guides (putty index) (13%) Photographs (22%)16. What percentage of the time, as the dental technician, do you have to decide on the type of material to be used as it is not specified by the dentist on the laboratory prescription?0-25% (56%) 26-50% (20%) 51-75% (16%) 76-100% (8%)17. During the fabrication of a fixed dental prosthesis, what is the most commonly used metal alloy?High gold content (8%) Low gold content (14%) Palladium alloy (26%) Base metal (52%)18. What is the most commonly requested material for the construction of an anterior crown?Metal-ceramic (69%) All-ceramic (29%) Metal-composite (2%) Metal-acrylic (0%)19. What is the most commonly requested material for the construction of a posterior crown?Metal only (19%) Metal-ceramic (71%) All-ceramic (8%) Metal-composite (2%)20. What percentage of the time therefore do you have to decide on the surface needed to be covered with metal or aesthetic veneering material because it has not been prescribed by the dentist?0-25% (57%) 26-50% (20%) 51-75% (11%) 76-100% (12%)

## Notes

**Refereed Paper**
